# Residual risk in cardiovascular and renal diseases and the potential role of aldosterone synthase inhibitors

**DOI:** 10.3389/fcvm.2026.1845339

**Published:** 2026-05-29

**Authors:** Kausik Umanath, Jamie P. Dwyer, Robert J. Mentz

**Affiliations:** 1 Division of Nephrology and Hypertension, Department of Internal Medicine, Indiana University School of Medicine, Indianapolis, IN, United States; 2 Division of Nephrology and Hypertension, Department of Internal Medicine, University of Utah Health, Salt Lake City, UT, United States; 3 Division of Cardiology, Department of Medicine, Duke University School of Medicine, Durham, NC, United States; 4 Duke Clinical Research Institute, Durham, NC, United States

**Keywords:** aldosterone, aldosterone synthase inhibitor, cardiovascular disease, chronic kidney disease, clinical trial, mineralocorticoid receptor, mineralocorticoid receptor antagonist

## Abstract

Renin-angiotensin-aldosterone system (RAAS) blockade has been a mainstay of therapy in cardiovascular and kidney disease for nearly 30 years. Despite the development of multiple drug classes designed to intervene along the RAAS pathway, the residual risk for the totality of adverse cardiorenal outcomes (cardiorenal disease) remains substantial. Dysregulation of aldosterone biosynthesis has pathophysiological consequences that are associated with cardiovascular and kidney damage. Aldosterone synthase inhibitors (ASi) provide a mechanism to decrease aldosterone production. These agents may help to reduce residual risk in cardiorenal disease. Preliminary evidence from ASi clinical trials is encouraging, with demonstrated efficacy in improving blood pressure control and decreasing albuminuria in chronic kidney disease. Their therapeutic potential in improving cardiorenal outcomes is being investigated further in ongoing phase III trials where ASi agents are given as monotherapy or with an SGLT2 inhibitor, and these data will be available over the coming months and years. ASi agents may be a new evidence-based addition to the cardiorenal drug armamentarium.

## Graphical Abstract

**Figure:**
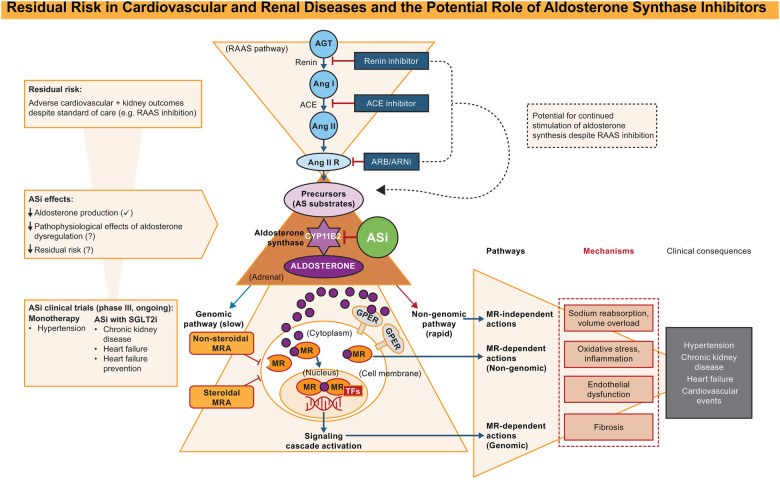
ACE, angiotensin-converting enzyme; AGT, angiotensinogen; Ang I, angiotensin I; Ang II, angiotensin II; Ang II R, angiotensin II receptor; ARB, angiotensin II receptor blocker; ARNi, angiotensin receptor-neprilysin inhibitor; AS, aldosterone synthase; ASi, aldosterone synthase inhibitor; CYP11B2, cytochrome P450 family 11 subfamily B member 2; GPER, G protein-coupled estrogen receptor; MR, mineralocorticoid receptor; MRA, mineralocorticoid receptor antagonist; RAAS, renin-angiotensin aldosterone system; SGLT2i, sodium glucose cotransporter-2 inhibitor; TFs, transcription factors. (Red T-bar represents inhibition.).

## 1 Introduction

The renin-angiotensin-aldosterone system (RAAS) is a critical regulator of blood volume, electrolyte balance, and systemic vascular resistance (1). Pathological activation or overactivation of the RAAS pathway has long been recognized as a key mediator of cardiovascular (CV) and kidney disease (hereafter referred to as cardiorenal disease), including CV events, heart failure (HF), and chronic kidney disease (CKD) progression (2–5). Multiple classes of drugs have been developed to intervene along the RAAS pathway, including angiotensin-converting enzyme inhibitors (ACEi), angiotensin II receptor blockers (ARB) (6), angiotensin receptor-neprilysin inhibitors (7, 8), direct renin inhibitors (9), and mineralocorticoid receptor antagonists [MRAs, comprising of steroidal and nonsteroidal (nsMRA) agents] (10). Other drug classes proven to improve cardiorenal outcomes include sodium glucose cotransporter-2 inhibitors (SGLT2i) (11, 12) and glucagon-like peptide-1 (GLP-1) receptor agonists (13, 14).

**Figure 1:**
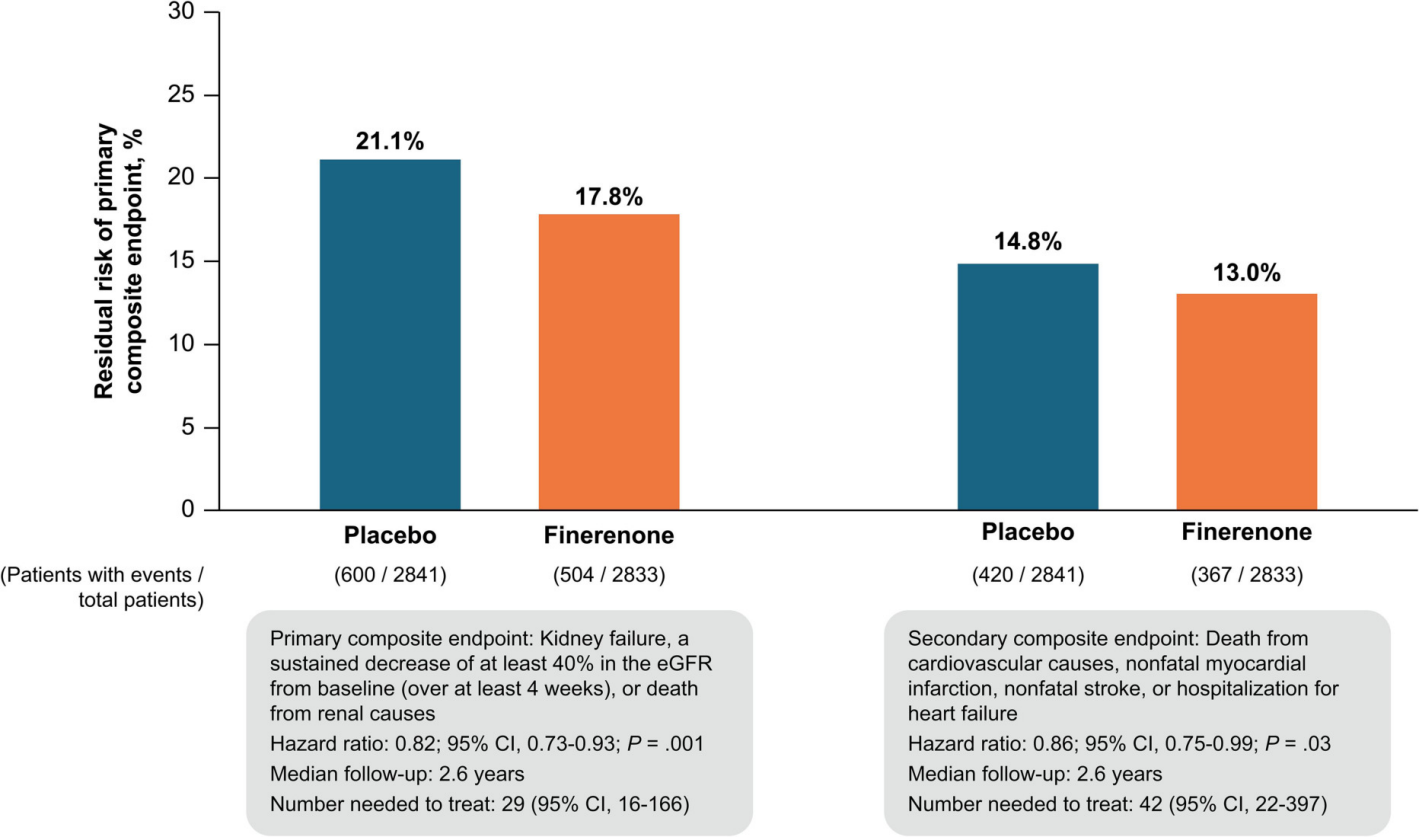
Evaluation of residual risk for the primary and secondary composite endpoint in the FIDELIO-DKD clinical trial (15). CI, confidence interval; eGFR, estimated glomerular filtration rate; FIDELIO-DKD, Finerenone in Reducing Kidney Failure and Disease Progression in Diabetic Kidney Disease.

**Figure 2:**
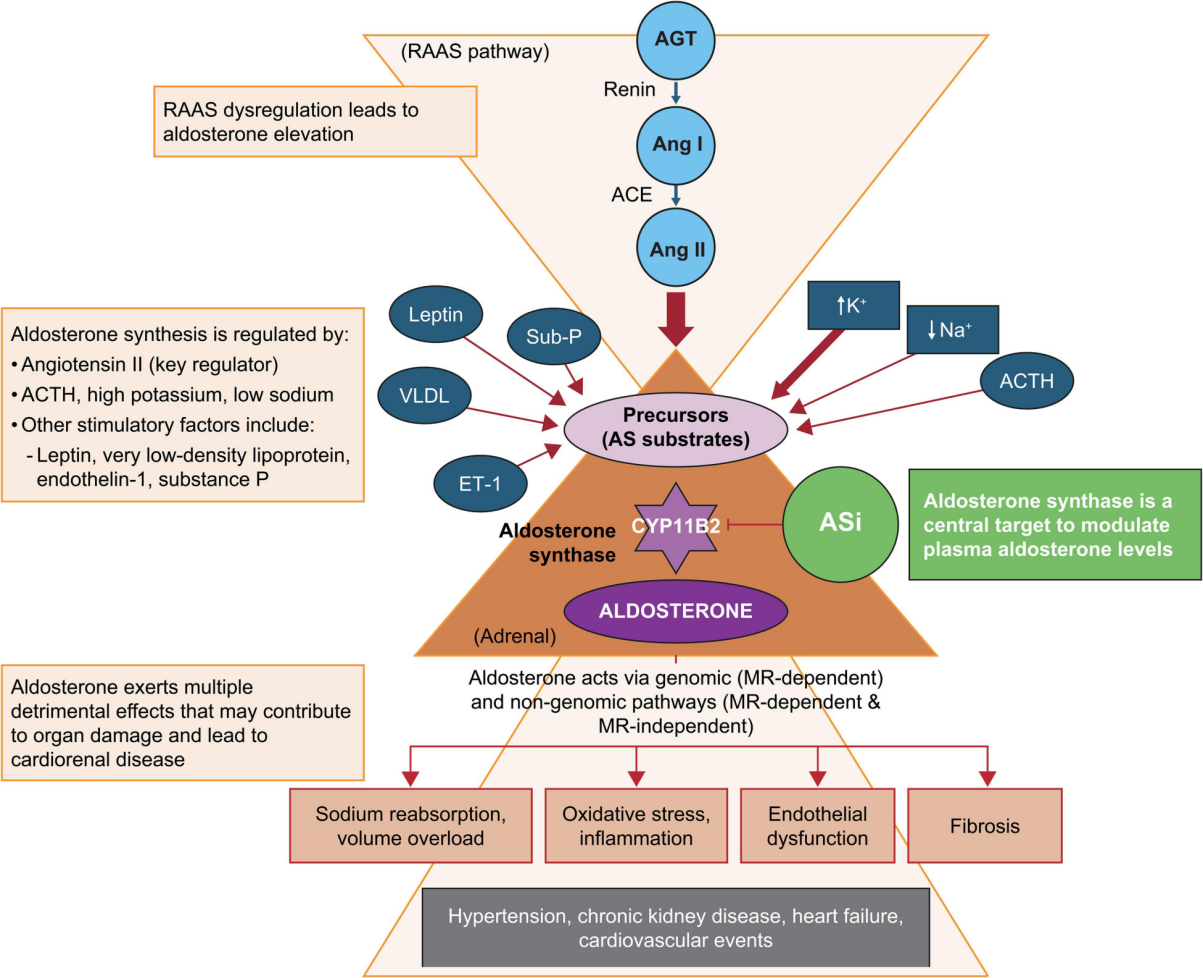
Aldosterone synthase: A central target for modulating aldosterone levels. RAAS dysregulation and other triggers lead to elevated aldosterone production, a key driver of cardiorenal disease. Aldosterone synthase is, therefore, a central target for modulating plasma aldosterone levels. ACE, angiotensin-converting enzyme; ACTH, adrenocorticotropic hormone; AGT, angiotensinogen; Ang I, angiotensin I; Ang II, angiotensin II; AS, aldosterone synthase; ASi, aldosterone synthase inhibitor; CYP11B2, cytochrome P450 family 11 subfamily B member 2; ET-1, endothelin-1; K^+^, potassium; MR, mineralocorticoid receptor; Na^+^, sodium; RAAS, renin-angiotensin aldosterone system; Sub-P, substance P; VLDL, very low-density lipoprotein.

Despite these various interventions, the residual risk for the totality of adverse CV and kidney outcomes remains substantial (where residual risk refers to the remaining risk of adverse outcomes, despite treatment with the standard of care [SoC]) (15, 16). The concept of residual risk is illustrated by data from the phase III FIDELIO-DKD (NCT02540993) trial, which assessed CKD progression and CV outcomes in patients with advanced CKD and type 2 diabetes (T2D) (15). Despite the significant benefits observed with finerenone, 17.8% of trial participants receiving finerenone experienced a primary endpoint after median follow-up of 2.6 years, and 13.0% of those receiving finerenone experienced a continuing risk of CV events (Figure 1) (15). Aldosterone synthase inhibitors (ASi) are a new drug class that provide a mechanism to decrease aldosterone production and may help to reduce residual risk in cardiorenal disease (Figure 2). Their therapeutic potential for this and other endpoints is currently being investigated (17–20). The aim of this review is to summarize the present state of development of ASi agents and discuss central aspects and considerations of ASi therapy with respect to their potential impact on the residual risk of adverse CV and kidney outcomes.

## 2 Search strategy and selection criteria

Literature was retrieved from PubMed and Google Scholar databases, with an emphasis placed on articles published from 2010 onwards, using Boolean searches for terms related to the following: aldosterone, cardiorenal disease, cardiovascular disease, chronic kidney disease, heart failure, residual risk, nonsteroidal mineralocorticoid receptor antagonist, steroidal mineralocorticoid receptor antagonist, finerenone, aldosterone synthase inhibitor (or inhibition), baxdrostat, dexfadrostat, lorundrostat, and vicadrostat (searches were limited to human data and English-language articles). The reference lists from retrieved articles were also considered. Additional relevant articles were included manually. Other relevant literature was obtained based on the personal knowledge and experience of the authors. Further data were obtained from the United States (US) National Institutes of Health ClinicalTrials.gov website, and from other websites pertaining to individual therapeutic agents of interest. The retrieved references were assessed by the authors for their relevance to the scope of this review, from which a final reference list was generated that formed the basis of this narrative review.

## 3 Aldosterone pathway: its role as a mediator of cardiorenal disease

**Figure 3:**
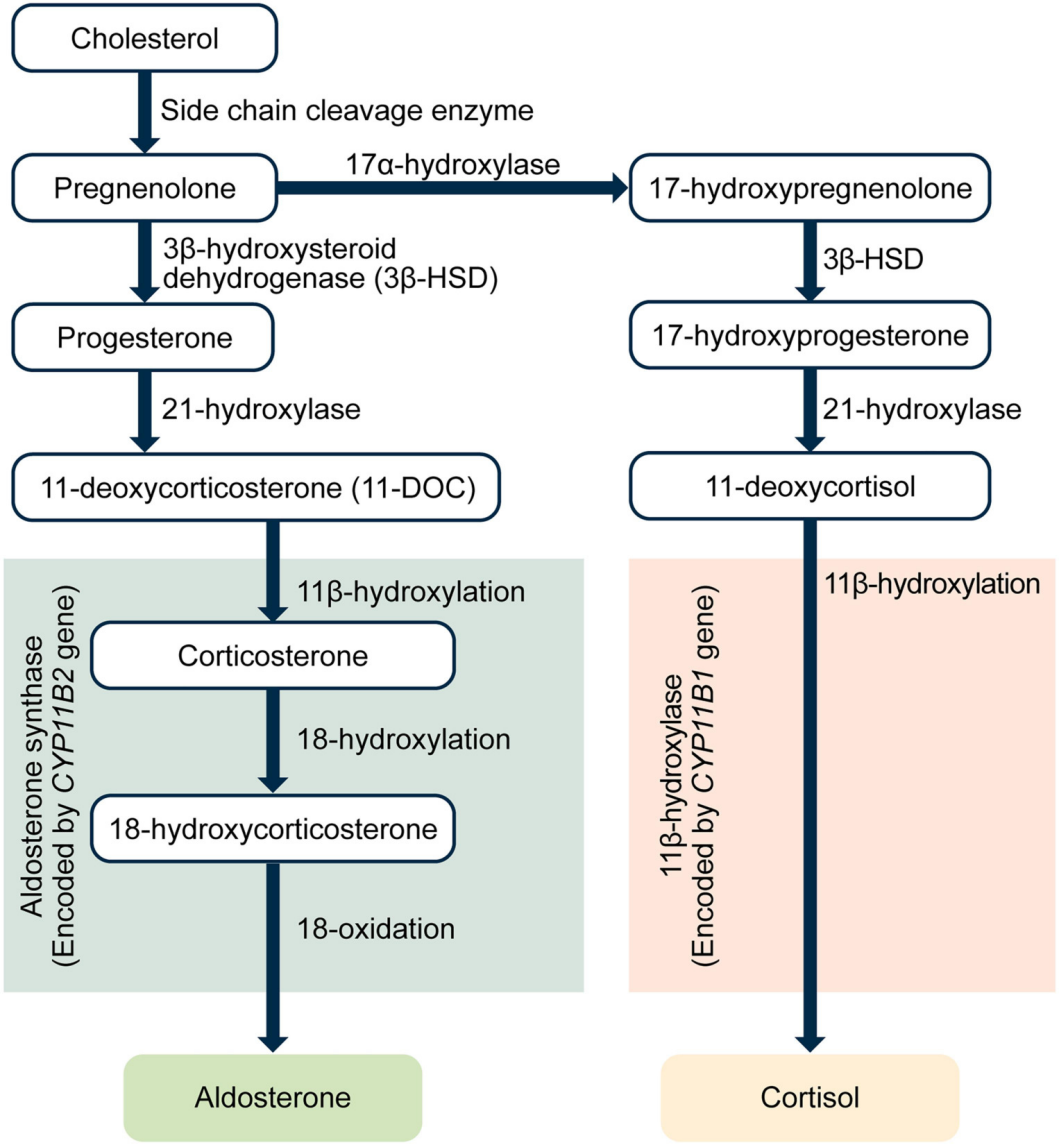
Biosynthesis of aldosterone in the adrenal gland (26). (From: Zhang M, Wu K, Long S, Jin X, Liu B. Prediction of pharmacokinetic/pharmacodynamic properties of aldosterone synthase inhibitors at drug discovery stage using an artificial intelligence-physiologically based pharmacokinetic model. Front. Pharmacol. 2025;16: 1578117. doi: 10.3389/fphar.2025.1578117. Used under CC BY 4.0.).

Aldosterone acts via the mineralocorticoid receptor (MR), which is encoded by the gene *Nuclear Receptor Subfamily 3 Group C Member 2* (*NR3C2*) (21). Aldosterone acts on tubular cells in the distal nephron, where it has a central role in electrolyte and fluid homeostasis (22). Aldosterone also targets a range of extra-renal sites where the MR is expressed, including cardiac and vascular tissues, the brain, adipose tissue, leukocytes, and erythrocytes (23). Aldosterone is produced primarily in the *zona glomerulosa* of the adrenal cortex via the enzyme aldosterone synthase, which is the protein product of gene *CYP11B2*, a member of the cytochrome P450 family (24). Adrenal aldosterone synthesis is regulated mainly by angiotensin II and blood potassium levels, with a lesser stimulatory effect from adrenocorticotropic hormone (ACTH) and other factors. Extra-adrenal sites of aldosterone synthesis have been identified, although their physiological and pathophysiological significance is not yet fully understood (25). Aldosterone synthase (CYP11B2) catalyzes the final 3 rate-limiting steps in aldosterone synthesis (Figure 3 26). Gene *CYP11B2* is structurally similar to gene *CYPB11B1*, and the latter encodes a key enzyme involved in the production of cortisol (11-beta-hydroxylase), a glucocorticoid synthesized in the *zona fasciculata* of the adrenal cortex. Genes *CYP11B2* and *CYP11B1* share 95% homology in their coding regions and 93% homology in their protein products (27). Historically, this has presented a major challenge in the development of ASi drugs as early candidates were hampered by limited specificity for the gene products of *CYP11B2* vs. *CYP11B1* and the resulting side effects caused by off-target activity (such as reduced glucocorticoid production) (28). Aldosterone exerts its effects via genomic and nongenomic pathways (22), and there is evidence of interplay between these pathways (29). In the genomic pathway, whose effects manifest over hours to days, aldosterone binds to MRs in the cell cytoplasm and is subsequently translocated to the nucleus, where it acts as a transcription factor by binding to the promoter regions of various genes. Nongenomic pathway effects are rapid (occurring in seconds to minutes) due to non-transcription-dependent transport activity at the cell membrane mediated via MR and by other types of receptor (ie, non-MR dependent; e.g., G protein-coupled estrogen receptor 1, various growth factor receptors, and angiotensin II receptor type 1), and may enhance genomic effects by functioning as feedback loops (29). Therapeutic interventions differ in their ability to suppress these pathways. ASi agents reduce aldosterone synthesis, thereby limiting ligand availability, and could broadly suppress both genomic and nongenomic signalling (17). In contrast, MRAs act downstream to block MR-dependent genomic effects but may incompletely inhibit nongenomic signalling (30), and often induce compensatory increases in circulating aldosterone. RAS inhibitors, on the other hand, act upstream in the renin–angiotensin system, lowering aldosterone indirectly and transiently, and are associated with incomplete pathway suppression (called “aldosterone breakthrough”) (31, 32).

The role of aldosterone in target organ damage to the heart and kidneys has been described extensively (17, 33–37), supported by data from mechanistic, preclinical, and clinical studies. Animal models demonstrated that excess aldosterone caused inflammatory, oxidative, and fibrotic effects in the heart, kidney, and blood vessels (38–44). Dysregulation of aldosterone and MR activation adversely affects various CV cell types (including cardiomyocytes, vascular smooth muscle cells, endothelial cells, inflammatory cells, and fibroblasts) and results in cardiac hypertrophy, cardiac remodeling, arrhythmias, atherosclerosis, inflammation, and cardiac fibrosis (34, 35, 45). As a mediator of kidney damage, aldosterone acts on kidney vessels, kidney cells, and infiltrating inflammatory cells via multiple mechanisms to promote kidney inflammation, injury, and fibrosis (17, 33, 35, 46). Aldosterone activation of the MR stimulates the generation of reactive oxygen species (ie, superoxide and hydrogen peroxide) that induce mitochondrial dysfunction and activate pro-inflammatory transcription factors, such as activator protein and nuclear factor kappa B (47, 48). Aldosterone also stimulates the expression of profibrotic molecules, such as transforming growth factor *β*1, plasminogen activator factor 1, endothelin, placental growth factor, connective tissue growth factor, and osteopontin (49–53). In a review of 200 publications of mechanistic studies, preclinical studies, clinical trials, and genetic association studies, data strongly supported an association between HF and dysregulated aldosterone/MR activation (34). The latter was also associated with atrial fibrillation and myocardial infarction, but less strongly (34). Patients with primary aldosteronism (PA) had a higher risk of adverse CV outcomes (including coronary artery disease, HF, left ventricular hypertrophy, atrial fibrillation, and stroke) that were independent of elevated blood pressure (BP) (54). Mitigation of adverse aldosterone effects in patients with PA, via adrenalectomy or treatment with MRA, significantly reduced the risk of CV events, mortality, and atrial fibrillation (55–58). Meta-analyses of MRA phase II and III trials showed that MRAs lowered CV mortality in patients with HF and reduced ejection fraction (HFrEF) (59, 60), mildly reduced ejection fraction (HFmrEF) (60), and preserved ejection fraction (HFpEF) (60). Data from the Chronic Renal Insufficiency Cohort (CRIC) study showed that higher serum aldosterone was associated with an increased risk of CKD progression that was independent of baseline kidney function or concomitant diabetes, whereby doubling of serum aldosterone concentration was linked to an 11% increased risk (37). The adverse effects of aldosterone may also be mediated by the phenomenon of “aldosterone breakthrough,” in which aldosterone levels return to (or exceed) pretreatment levels during RAAS inhibitor therapy (31). This is distinct from “aldosterone escape,” which describes the process whereby the kidneys excrete excess sodium and prevent severe volume expansion despite elevated mineralocorticoid levels (31). Patients who develop aldosterone breakthrough may have a worse clinical prognosis than those who do not, due to its association with faster deterioration in kidney function and more severe proteinuria (61–63). Blocking aldosterone production may address both the genomic and nongenomic effects of elevated aldosterone (17) and may prevent the occurrence of aldosterone breakthrough.

## 4 Mineralocorticoid receptor antagonists: existing agents with anti-aldosterone effects

Spironolactone, a steroidal MRA, was the first anti-aldosterone drug to be approved by the US Food and Drug Administration (FDA) and was marketed in the 1960s as a potassium-sparing diuretic for use in patients with edema, PA, and hypertension. However, spironolactone is a nonselective agent with affinity for androgen and progesterone receptors, which contributed to problematic side effects such as gynecomastia and erectile dysfunction in males, and menstrual irregularities in females. Eplerenone is a second-generation MRA that also possesses a steroidal structure but has increased MR selectivity and a more favorable side effect profile and gained FDA approval in 2002. Preclinical data demonstrated cardiorenal protective effects of spironolactone, which were later confirmed for spironolactone and eplerenone via key clinical trials in patients with HF (59, 60, 64) and kidney disease (65–67). A third-generation MRA, finerenone, was subsequently developed with a nonsteroidal structure and greater affinity for the MR (Table 1) (17, 18, 68–78). Finerenone received initial FDA approval in 2021 for the risk reduction of adverse cardiorenal outcomes in adults with CKD associated with T2D, with data from subsequent pivotal randomized clinical trials further demonstrating efficacy in CKD in T2D (15, 79–81), as well as in HFmrEF and HFpEF (across a range of higher ejection fractions) (16, 60). Other nsMRA agents include esaxerenone, which was approved in Japan in 2019 to treat primary hypertension (82), and ocedurenone, which was terminated during its phase III trial in participants with hypertension for failing to meet the primary endpoint (83). More recently, nsMRA/SGLT2i combination therapy was investigated in a phase II trial (CONFIDENCE; NCT05254002), in which finerenone plus empagliflozin led to greater reductions in albuminuria and BP in people with CKD and T2D than SGLT2i monotherapy or placebo (84). Lastly, balcinrenone (AZD9977), a novel nonsteroidal selective MR modulator (85), is currently in clinical development for use in HF and CKD as a combination therapy with dapagliflozin (86, 87).

**Table 1:** Comparison of steroidal MRA, nsMRA, and ASi agents.

	Steroidal MRA (68, 69)	nsMRA (68–70)	ASi (18)
Examples	Spironolactone (first generation); Eplerenone (second generation)	Finerenone	Baxdrostat, dexfadrostat, lorundrostat, vicadrostat
Mechanism	Antagonizes MR; prevents aldosterone binding Spironolactone: Potent, nonselective; partial agonist Eplerenone: Less potent but more selective; partial agonist	Antagonizes MR; prevents aldosterone binding Potent, selective; inverse agonist (ie, reduces MR cofactor recruitment even in the absence of aldosterone); suppresses transcription of genes induced by aldosterone	Inhibits aldosterone synthase; reduces aldosterone production
Distribution (rodent tissue)	Kidney > heart Spironolactone: 6:1 Eplerenone: 3:1	Kidney=heart; 1:1	Details are not yet available
Pharmacokinetics	Spironolactone: Prodrug, multiple active metabolites with long half-lives (>20 h) Eplerenone: No active metabolites; short half-life (4–6 h)	No active metabolites; short half-life (2–3 h)	Baxdrostat: multiple active metabolites (71) Half-lives: Baxdrostat (parent compound): 26–31 h (71) Dexfadrostat: 9.5–11 h (72) Lorundrostat: 10–12 h (73) Vicadrostat: 4–6 h (74)
Uses	Marketed: Spironolactone: HFrEF, HTN Eplerenone: HFrEF, HTN	Marketed: Diabetic nephropathies (risk reduction of disease progression and adverse CV outcomes in CKD associated with T2D) HFpEF (risk reduction of CV death, hospitalization for HF, and urgent HF visits)	Not yet marketed: in clinical development
		Phase III trials: CKD; HF*; PA (*MOONRAKER program (76); FINEARTS-HF, REDEFINE-HF, CONFIRMATION-HF, FINALITY-HF)	Phase III trials: Baxdrostat: HTN Lorundrostat: HTN Baxdrostat/dapagliflozin: CKD, HF prevention Vicadrostat/empagliflozin: CKD, HF, CV risk reduction (Dexfadrostat: PA, phase II trial)
Side effects	Hyperkalemia risk Off-target effects Spironolactone: Off-target effects on sex hormone receptors (gynecomastia, impotence, amenorrhea) Eplerenone: Fewer off-target effects	Hyperkalemia risk; lower discontinuation rates due to hyperkalemia vs spironolactone (77)	Hyperkalemia risk; this is expected to be reduced when given with SGLT2i
Other effects of note	Risk of counter-regulatory increase in renin and aldosterone that may: (i) Overcome MR blockade, and (ii) Stimulate MR-independent aldosterone effects (18)	Effects on plasma aldosterone are not widely reported	Reduction in plasma aldosterone (78)
Benefits	Control of genomic aldosterone effects Partial effects on nongenomic aldosterone pathway	Control of genomic aldosterone effects Partial effects on nongenomic aldosterone pathway	Control of genomic and nongenomic aldosterone effects could be expected (17)
Knowledge gaps		Efficacy to be determined for: CKD patients without diabetes CKD patients with type 1 diabetes PA	Long-term efficacy and safety data are awaited; Phase III trials are underway

ASi, aldosterone synthase inhibitor; CKD, chronic kidney disease; CV, cardiovascular; HF, heart failure; HFpEF, heart failure with preserved ejection fraction; HTN, hypertension; MR, mineralocorticoid receptor; MRA, mineralocorticoid receptor antagonist; ns, nonsteroidal; PA, primary aldosteronism; rEF, reduced ejection fraction; SGLT2i, sodium glucose cotransporter-2 inhibitor.

Steroidal MRAs, particularly spironolactone, are associated with an increased risk of hyperkalemia (HK) (88), which limits their widespread use in clinical practice. The potential risk of HK associated with RAAS inhibitor therapy may lead to dose reduction or discontinuation, with treatment interruption negatively impacting patient outcomes (89). For example, although discontinuation of MRA following HK is associated with reduced risk of HK recurrence, the subsequent risk of adverse CV events or death may be increased (90). Comparison of HK rates from different trial populations treated with steroidal MRA vs. nsMRA suggests that a lower incidence of HK could be expected with finerenone (77); however, data from head-to-head comparison trials are limited, and real-world evidence and data from ongoing phase III trials of MRAs are awaited (91). In a prespecified analysis of the FINEARTS-HF trial (NCT04435626) (16), the risk of HK (potassium >5.5 mmol/L) was higher in patients treated with finerenone compared with those receiving placebo across all ejection fraction groups (an approximately 2-fold increase vs. placebo) (92). A *post hoc* analysis from the FIDELIO-DKD trial also reported approximately 2-fold higher rates of HK in participants receiving finerenone vs. those receiving placebo (after 2.6 years' median follow-up, respectively: mild HK [>5.5 mmol/L]; 21.4% vs. 9.2%; moderate HK [>6.0 mmol/L]; 4.5% vs. 1.4%) (93). The impact of HK was diminished by potassium monitoring and HK management strategies (93).

## 5 Aldosterone synthase inhibitors: new agents that block aldosterone synthesis

Osilodrostat (LCI699) was the first ASi to be developed for use in humans (94); however, its selectivity in inhibiting the gene products of *CYP11B2* vs. *CYP11B1* was limited *in vitro*, and doses greater than 1 mg inhibited cortisol secretion (basal and ACTH-stimulated) (95). Consequently, it was repurposed and developed as a treatment for Cushing's disease (96, 97). Next-generation ASis were developed with greater selectivity for the gene product of CYP11B2 vs. CYP11B1, and several agents are in the advanced stages of clinical development. These include baxdrostat (CIN-107), dexfadrostat (DP-13), lorundrostat (MLS-101), and vicadrostat (BI 690517). Data from ASi key clinical trials are summarized in Table 2 (18, 71–74, 78, 98–117). Phase II and III clinical trial data have demonstrated the efficacy and safety of ASi monotherapy in BP reduction in people with treatment-resistant hypertension (R-HTN) and uncontrolled hypertension (101, 102, 106, 108, 112). Baxdrostat and lorundrostat produced placebo-adjusted systolic BP reductions of approximately 9 to 12 mmHg (least-squares mean difference) in their phase III trials (BaxHTN, *N* = 796 and Launch-HTN, *N* = 1083, respectively) when added to background therapy (108, 112). A further baxdrostat phase III trial in people with R-HTN (Bax24, *N* = 218) demonstrated a significant placebo-adjusted reduction in ambulatory 24-hour average SBP of 14.0 mmHg (95% CI −17.2, −10.8; *p* < 0.0001) after 12 weeks (109).

**Table 2:** Summary of the Key characteristics of the main ASi agents in clinical development.

	Baxdrostat	Dexfadrostat	Lorundrostat	Vicadrostat
Structure https://pubchem.ncbi.nlm.nih.gov	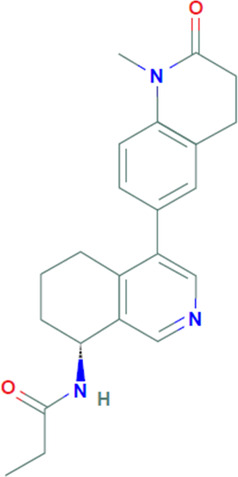	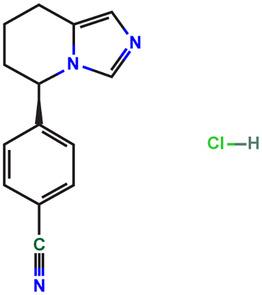	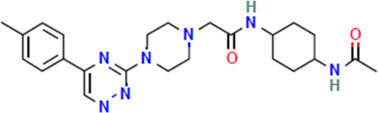	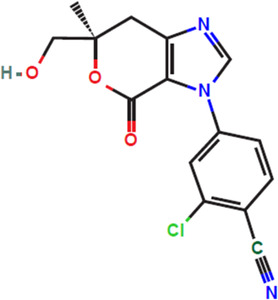
Sponsor's compound code	CIN-107	DP-13	MLS-101	BI 690517
Selectivity (CYP11B2^a^: CYP11B1^b^)	100: 1 (98)	9: 1 (18)	374: 1 (18)	250: 1 (99)
Plasma half-life, h	26-31 (71)	9.5-11 (72)	10-12 (73)	∼4–6 (74)
Phase II trials (CTG identifier; indication; status; primary completion)				
HTN	NCT06336356; U-HTN; completed (Dec 2024)	*(No trials listed for HTN)*	NCT06785454; OSA, HTN; recruiting (expected completion Q1 2026)	*(No trials listed for HTN)*
	NCT05137002 (HALO); U-HTN; completed (Sept 2022) (100)		NCT05769608 (Advance-HTN); HTN; completed (Jan 2025) (105)	
	NCT05459688 (HALO-OLE); U-HTN; completed (November 2023)			
	NCT04519658 (BrigHTN); R-HTN; completed (June 2022) (101)		NCT05001945 (TARGET); U-HTN; completed (October 2022) (106)	
CKD	NCT05432167 (FigHTN); CKD and U-HTN; completed (May 2024) (102) NCT07222917 (BaxDuo-Baltic); prestart (completion expected May 2027)	*(No trials listed for CKD)*	NCT06150924 (Explore-CKD); CKD; completed (May 2025) (103)	NCT05182840; CKD; completed (July 2023) (78) NCT06926660 (EASi-SYNCH); CKD (completion expected August 2026)
PA	NCT04605549 (SPARK); PA; completed (October 2024)	NCT04007406; PA; completed (May 2022) (104)	*(No trials listed for PA)*	*(No trials listed for PA)*
SBP lowering effect, mmHg	Phase II, patients with R-HTN (BrigHTN) (101): (*N* = 248) At week 12, 2 mg −20.3; 1 mg −17.5; vs PBO −9.4 2 mg vs PBO: −11.0 (95% CI, −16.4 to −5.5; *P* < .001) Phase II, patients with CKD and U-HTN (FigHTN) (102): (*N* = 195) At week 26, mean PBO-corrected change for baxdrostat pooled group −8.1 (95% CI, −13.4 to −2.8); *P* = .003; low-dose (0.5 to 1 mg) −9.0 (−15.1 to −2.9); *P* = .004; high-dose (2 to 4 mg) −7.2 (−13.2 to −1.2); *P* = .02	Phase II, patients with PA (104): (*N* = 35) Combined dose group over treatment period, LSM change in aSBP: −10.7 (95% CI, −13.6 to −7.9; *P* < .0001)	Phase II, patients with U-HTN (TARGET) (106): (*N* = 200) 100 mg −14.1; 50 mg −13.2; vs PBO −4.1 LSM difference 100 mg vs PBO: −7.8 (90% CI, −14.1 to −1.5; *P* = .04); 50 mg vs PBO: −9.6 (90% CI, −15.8 to −3.4; *P* = .01) Phase IIb, patients with R- and U-HTN (Advance-HTN) (105): (*N* = 295; 50 mg [stable dose group] *N* = 94; 100 mg [dose-adjustment group] *N* = 96; PBO *N* = 95) After 12 weeks, LSM change 24-h average SBP: 50 mg −15.4; 100 mg −13.9; PBO −7.4 PBO-adjusted change in BP vs 50 mg: −7.9 (97.5% CI, −13.3 to −2.6); PBO-adjusted change in BP vs 100 mg: −6.5 (97.5% CI, −11.8 to −1.2)	Phase II, patients with CKD (78): (*N* = 586; monotherapy *N* = 298; with EMPA 10 mg *N* = 288) (Monotherapy) 20 mg −4.94; 10 mg −0.72; PBO 1.09 (With EMPA 10 mg) 20 mg −5.78; 10 mg −5.34; PBO 2.47 (Monotherapy) 20 mg vs PBO: −6.03 (95% CI, −12.44 to 0.38); 10 mg vs PBO: −1.81 (95% CI, −8.10 to 4.48), (With EMPA 10 mg) 20 mg vs PBO: −8.25 (95% CI, −13.40 to −3.09); 10 mg vs PBO: −7.81 (95% CI, −13.69 to −1.92)
UACR reduction effect, %	Phase II, patients with R-HTN (107): (*N* = 275) 2 mg −33.7; 1 mg −19.9 vs PBO −2.8 2 mg vs PBO: −31.8 (95% CI, −49.1 to −8.6); *P* = .011 1 mg vs PBO: −17.6 (95% CI, −38.5 to 10.4); *P* = .196	*(No data)*	Phase II, patients with CKD and U-HTN (Explore-CKD) (103): (*N* = 59; lorundrostat 25 mg followed by PBO [L-P], *n* = 30; PBO followed by lorundrostat [P-L], *n* = 29) GM (PBO-adjusted) −25.6% (90% CI, −35.8 to −13.7; *P* = .0015)	Phase II, patients with CKD (78): (*N* = 586; monotherapy *N* = 298; with EMPA 10 mg *N* = 288) (Monotherapy) 20 mg −37; 10 mg −39; PBO −3 (With EMPA 10 mg) 20 mg −40; 10 mg −46; PBO −11 (Monotherapy) 20 mg vs PBO: −35 (95% CI, −51 to −14) 10 mg vs PBO: −37 (95% CI, −52 to −18) (With EMPA 10 mg) 20 mg vs PBO: −33 (95% CI, −47 to −17) 10 mg vs PBO: −40 (95% CI, −52 to −24)
Phase III trials (CGT identifier; indication; status (study completion))				
HTN	NCT06344104 (BaxAsia); U-HTN and R-HTN; active (expected completion Q2 2026)	*(No phase III trials listed for HTN)*	NCT05968430; HTN-OLE; active (expected completion Q4 2026)	*(No phase III trials listed for HTN)*
	NCT06034743 (BaxHTN); U-HTN and R-HTN; completed (Oct 2025) (108)		NCT06153693 (Launch-HTN); U-HTN and R-HTN; completed (Jan 2025) (112)	
	NCT06168409 (Bax24); R-HTN; completed (results expected 4Q 2025) (109)			
CKD	NCT06268873 (BaxDuo-ARCTIC); CKD and HTN; recruiting (expected completion Q1 2028) (110)	*(No phase III trials listed for CKD)*	*(No phase III trials listed for CKD)*	NCT06531824 (EASi-KIDNEY); CKD; recruiting (expected completion Q3 2028) (113)
	NCT06742723 (BaxDuo-PACIFIC); CKD and HTN; recruiting (expected completion Q2 2030) (111)			
HF	*(No phase III trials listed for HF)*	*(No phase III trials listed for HF)*	*(No phase III trials listed for HF)*	NCT06424288 (EASi-HF Preserved); HF; recruiting (expected completion Q2 2028) (115) NCT06935370 (EASi-HF Reduced) HF; recruiting (expected completion Q1 2029) (116)
HF prevention	NCT06677060 (Prevent-HF); T2D, HTN, and CVD; prestart; recruiting (expected completion Q4 2029) (114)	*(No phase III trials listed for HF prevention)*	*(No phase III trials listed for HF prevention)*	NCT07064473 (EASi-PROTKT); T2D, HTN, and CVD; recruiting (expected completion Q4 2029) (117)
SBP lowering effect, mmHg	Phase III, patients with R- and U-HTN (BaxHTN) (108): *N* = 796 (1:1:1 to 1 mg OD, 2 mg OD, or PBO) At week 12, LSM difference vs placebo: −8.7 (95% CI, −11.5 to −5.8) with 1 mg and −9.8 (95% CI, −12.6 to −7.0) with 2 mg; *P* < .001 for both comparisons	—	Phase III, patients with R- and U-HTN (Launch-HTN) (112): *N* = 1083 (1:2:1 to PBO, 50 mg OD, or up-titration to 100 mg OD) For 50 mg dose, auto-office SBP, at week 6: LSM difference vs placebo: −9.1 (95% CI; −13.3 to −4.9); *P* < .001; at week 12, LSM difference vs placebo: −11.6 (95% CI; −16.3 to −7.0); *P* < .001	—

AS, aldosterone synthase; aSBP, ambulatory 24-h systolic blood pressure; auto-office, automated office reading; CKD, chronic kidney disease; CTG, ClinicalTrials.gov; CVD, cardiovascular disease; EMPA, empagliflozin; GM, geometric mean; HF, heart failure; HTN, hypertension; LSM, least-squares mean; OSA, obstructive sleep apnea; PA, primary aldosteronism; PBO, placebo; Q1/2/3/4, first/second/third/fourth quarter (of the year); R-HTN, (treatment) resistant hypertension; SBP, systolic blood pressure; UACR, urine albumin-to-creatinine ratio; U-HTN, uncontrolled hypertension.

a *CYP11B2,* the gene encoding aldosterone synthase (which catalyzes conversion of 11-deoxycorticosterone to corticosterone, to 18-hydroxycorticosterone, and then to aldosterone).

b *CYP11B1,* the gene encoding 11*β*-hydroxylase (which catalyzes conversion of 11-deoxycortisol to cortisol).

Several clinical trials are evaluating ASi given with SGLT2i therapy added to SoC. A phase II trial in people with CKD demonstrated dose-dependent reductions in albuminuria when vicadrostat was given as monotherapy and with empagliflozin, alongside background renin-angiotensin system inhibition (78). This study (*N* = 587, of whom 298 received vicadrostat with empagliflozin) suggested an additive efficacy for vicadrostat plus empagliflozin (78). A phase III trial program is underway to evaluate the efficacy and safety of vicadrostat given with empagliflozin in improving cardiorenal outcomes in people with CKD and HF, and includes EASi-KIDNEY (planned enrollment [PE]: *N* = 11,000) (113), EASi-HF Preserved (PE: *N* = 6000) (115), EASi-HF Reduced (PE: *N* = 4200) (116), and EASi-PROTKT (PE: *N* = 11,800) (117) trials. Similarly, a phase III program to investigate the effect of baxdrostat with dapagliflozin in populations with hypertensive kidney disease and CVD/HF risk is in progress, and it includes BaxDuo-PACIFIC (PE: *N* = 5000) (111), BaxDuo-ARCTIC (PE: *N* = 2500) (110), and Prevent-HF (PE: *N* = 11,300) (114) trials. In both EASi and BaxDuo trial programs, the ASi plus SGLT2i therapy will be tested against active treatment (ie, SGLT2i monotherapy plus SoC) rather than against placebo (ie, placebo plus SoC). Overall participant numbers in these trials are considerably larger than those of the ASi monotherapy trials, suggesting that most clinical data on ASi agents will come from studies of ASi administered with SGLT2i. Co-administration of ASi and SGLT2i may be expected to enhance cardiorenal efficacy, given that SGLT2i have proven cardiorenal protective efficacy and safety in people with CKD (118) and are known to improve cardiac and kidney outcomes in people with HF and CKD (with or without T2D), respectively (119–121). This combination is further supported by the potential ability of SGLT2i to mitigate the risk of serious HK (serum potassium ≥6.0 mmol/L) in people with CKD and T2D (122). This is important given that ASi agents have the potential to increase serum potassium concentrations, as would be expected from their mechanism of action. A meta-analysis of ASi randomized controlled trials (RCT; 7 RCT; *N* = 1440) revealed an increased risk of HK vs. placebo (relative risk, 2.5; 95% CI, 1.2 to 5.4; *P* < .02; I^2^ = 0%), with HK occurring in 6.2% of ASi-treated patients vs. 1.2% of the placebo group; however, the increase in mean plasma potassium concentration was small (0.3 mmol/L for ASi vs. 0.0 mmol/L for placebo) (123). A further ASi meta-analysis (5 RCT; *N* = 2456) reported no increase in moderate-to-severe HK (≥6.0 mmol/L) for ASi at standard doses vs. placebo (odds ratio, 3.19; 95% CI, 0.79 to 12.95; *P* = .104), although a slight increase was associated with high-dose ASi regimens (odds ratio 4.43; 95% CI, 1.10 to 17.82; *P* = .036) (124). Evidence from an ASi network meta-analysis (6 RCT; *N* = 2149) suggested that most adverse events were mild, serious adverse events were infrequent, and no drug-related mortalities were reported (125). ASi agents have generally shown favorable safety profiles (125); however, results from large, long-term phase III RCTs are awaited.

## 6 Conclusions

Preliminary evidence suggests the ASi drug class may offer additional benefits for cardiorenal disease protection on top of those from existing therapies that act along the RAAS pathway. ASi monotherapies show promise for BP control, and ongoing clinical trials of ASi with SGLT2i therapies will elucidate further outcome benefits in CV and renal diseases. Analysis of data from the multiple phase III trials that are in progress is needed to confirm long-term safety and efficacy in wider patient populations (18). Head-to-head trials may be needed to compare ASi with nsMRA (18). The differing roles of combination therapy with ASi vs. nsMRA agents for cardiorenal protection in real-world practice also need to be distinguished (126). Therapy in the cardiorenal arena has changed dramatically in the past decade with the publication of multiple trials showing efficacy of SGLT2i, GLP-1 agonists, and nsMRAs; however, questions remain, including which combinations of these agents will reduce risk optimally and in which patient populations. Nevertheless, ASi agents — particularly when given with SGLT2i — appear well on the way to providing an additional drug class to combat the significant burden of cardiorenal disease outcomes.
